# Dr. Herbert Paris Sheldon

**Published:** 1912-11

**Authors:** 


Dr. Herbert Paris Sheldon, Buffalo 1879, died at his home in Scotts Bluff, Neb., Oct. 3, from broncho-pneumonia, aged 57.

